# Parathyroidectomy Associates with Reduced Mortality in Taiwanese Dialysis Patients with Hyperparathyroidism: Evidence for the Controversy of Current Guidelines

**DOI:** 10.1038/srep19150

**Published:** 2016-01-13

**Authors:** Li-Chun Ho, Shih-Yuan Hung, Hsi-Hao Wang, Te-Hui Kuo, Yu-Tzu Chang, Chin-Chung Tseng, Jia-Ling Wu, Chung-Yi Li, Jung-Der Wang, Yau-Sheng Tsai, Junne-Ming Sung, Tainan RENal Disease Study (TRENDS) group, Junne-Ming Sung, Jung-Der Wang, Chung-Yi Li, Chin-Chung Tseng, Yu-Tzu Chang, Te-Hui Kuo, Hsi-Hao Wang, Li-Chun Ho, Jia-Ling Wu, Chih-Cheng Hsieh, Miao-Fen Yen, Hung-Lien Wu, Ping-Yu Chen, Wen-Huang Li, Wei-Ting Chang

**Affiliations:** 1 Graduate Institute of Clinical Medicine, National Cheng Kung University, Tainan; 2 Division of Nephrology, Department of Internal Medicine, E-Da Hospital/I-Shou University, Kaohsiung; 3 Department of Internal Medicine, National Cheng Kung University Medical College and Hospital, Tainan; 4 Graduate Institute of Public Health, National Cheng Kung University, Tainan; 5 Department of Public Health, National Cheng Kung University College of Medicine, Tainan; 6 Department of Public Health, College of Public Health, China Medical University, Taichung; 7 Department of Occupational and Environmental Medicine, National Cheng Kung University Hospital, Tainan, Taiwan; 8 Department of Nursing, National Cheng Kung University Hospital, Tainan,; 9 Department of Nutrition, National Cheng Kung University Hospital, Tainan,; 10 Department of Internal Medicine, Chi-Mei Medical Center,, Tainan, Taiwan

## Abstract

Parathyroidectomy is recommended by the clinical guidelines for dialysis patients with unremitting secondary hyperparathyroidism (SHPT). However, the survival advantage of parathyroidectomy is debated because of the selection bias in previous studies. To minimize potential bias in the present nationwide cohort study, we enrolled only dialysis patients who had undergone radionuclide parathyroid scanning to ensure all patients had severe SHPT. The parathyroidectomized patients were matched with the controls based on propensity score for parathyroidectomy. Mortality hazard was estimated using multivariate Cox proportional hazard models adjusting for comorbidities before scanning (model 1) or over the whole study period (model 2). Our results showed that among the 2786 enrolled patients, 1707 underwent parathyroidectomy, and the other 1079 were controls. The crude mortality rates were lower in the parathyroidectomized patients than in the controls. In adjusted analyses for the population matched on propensity score, parathyroidectomy was associated with a significant 20% to 25% lower risk for all-cause mortality (model 1: hazard ratio 0.76, 95% confidence interval 0.61 to 0.94; model 2: hazard ratio 0.80, 95% confidence internal 0.64 to 0.98). We concluded that parathyroidectomy was associated with a reduced long-term mortality risk in dialysis patients with severe SHPT.

Parathyroid hyperplasia-related secondary hyperparathyroidism (SHPT) is common in dialysis patients^1^. Increased risk of death has been associated with serum levels of intact parathyroid hormone (iPTH) in excess of 600 pg/mL^2^,^3^. Conventional medical treatments, such as phosphate binders and vitamin D sterols, are seldom effective when parathyroid glands have progressed from diffuse hyperplasia to nodular hyperplasia^4^. Therefore, the National Kidney Foundation Kidney Disease Outcome Quality Initiative (NKF-K/DOQI) guidelines recommend surgical parathyroidectomy (PTx) for dialysis patients with persistent serum iPTH >800 pg/mL, especially when SHPT has resulted in hypercalcemia and/or hyperphosphatemia that are refractory to medical therapy^5^. Although the introduction of calcimimetics in 2004 appears to have caused a reduction in PTx rates^6^,^7^, the position of PTx as the principal therapy for unremitting SHPT seems unshaken because randomized controlled trials have failed to show definite effects of calcimimetics on all-cause and cardiovascular mortality^8^,^9^.

However, the recommendation of PTx in the guidelines is not based on solid evidence either. No randomized controlled trial has been conducted to compare the outcomes of medical and surgical therapy for SHPT. The largest retrospective cohort study, comparing 4558 dialysis patients undergoing PTx with the same number of matched controls not undergoing PTx, did show a lower relative risk of death in the PTx group^10^, yet the strength of this finding was potentially confounded by not matching for serum iPTH level and comorbidity, the two factors that are most likely to affect the clinical decision for PTx^11^,^12^,^13^. Other factors that are associated with the incidence of PTx and hence should be controlled in a cohort study include age, sex, dialysis duration, dialysis modality, renal transplantation, serum calcium, phosphate, and hemoglobin levels^7^,^11^,^12^,^13^,^14^. Comprehensive control over all risk factors is almost impossible for small observational studies, which is probably the reason for the conflicting results yielded from several studies addressing the issue of PTx outcomes in dialysis patients^15^,^16^,^17^,^18^,^19^.

The present study is a nationwide cohort study comparing the rates of all-cause mortality among dialysis patients undergoing PTx with those of a non-PTx control group. To minimize potential selection bias, we enrolled only individuals who had received radionuclide parathyroid imaging to ensure all patients had severe SHPT. We also aimed to control for confounding by appropriate matching techniques and multivariate adjustment in the statistical analyses.

## Results

**Figure 1:**
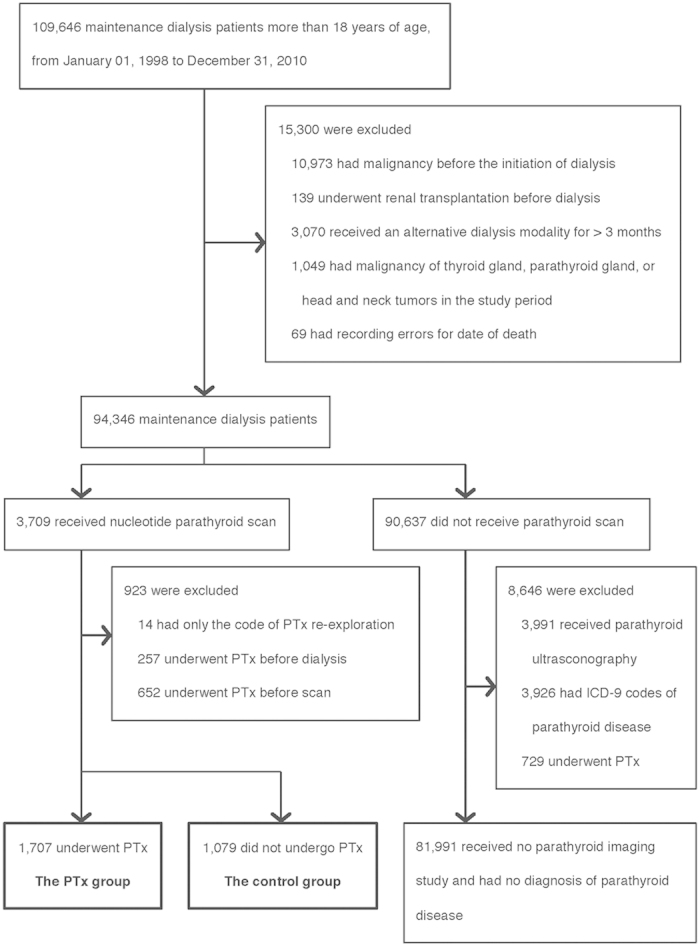
Patient selection. The aim of the selection is to identify maintenance dialysis patients who had received radionuclide parathyroid imaging, and hence are regarded as having severe secondary hyperparathyroidism. The reasons for exclusion are listed in the box. PTx, parathyroidectomy.

The flow diagram of patient selection is shown in Fig. 1. The final study population consisted of 2786 hemodialysis (HD) or peritoneal dialysis (PD) patients who had received radionuclide parathyroid scanning in the study period from January 1, 1998 to December 31, 2010. Among them, 1707 (61.3%) patients who had undergone PTx after scanning were classified as the PTx group. The other 1079 (38.7%) patients who did not undergo PTx were classified as the control group. In the PTx group, 183 (10.7%) patients underwent auto-transplantation of parathyroid gland (supplementary information).

The average time for the dialysis patients to undergo their first parathyroid scan was 4.61 ± 2.98 years after the initiation of dialysis, at a mean age of 54.0 ± 12.5 years old (Table 1). In comparison with the controls before matching, the PTx patients tended to be younger; with a lower proportion of males; receiving HD rather than PD; on regular dialysis for a longer duration; and less likely to have comorbidities including diabetes mellitus, hypertension, and cardiovascular diseases (Table 1). The differences in the distributions of the above variables between the two groups were largely balanced after matching, except that the proportions of hyperlipidemia, peripheral vascular disease, and gastrointestinal bleeding were higher (standardized difference >0.10) in the matched PTx group (Table 1).

Among the 2786 enrolled patients with parathyroid scan data, 492 died during the study period. Before matching, the PTx group had a longer mean follow-up time (3.37 ± 2.47 years vs. 2.62 ± 2.32 years) but smaller number of deceased cases (234 vs. 258 deaths) than the control group, which resulted in a lower incidence rate of death (407/10,000 person-years vs. 914/10,000 person-years). The trend of difference between the two groups was reduced but not reversed after the matching process (Table 2). The mortality rates of parathyroidectomized patients and propensity score-matched controls were 470/10,000 person-years and 848/10,000 person-years respectively (Table 2).

Cox proportional hazard model was used to overcome the residual differences in confounding after propensity score matching. It indicated that PTx was associated with a reduction of all-cause mortality rate in the matched population (hazard ratio = 0.76; 95% C.I. 0.61–0.94) after adjustments for age, sex, dialysis modality, dialysis duration, and comorbidities before parathyroid scan (Table 3, model 1). The survival benefit of PTx remained when the comorbidities before and after parathyroid scan were adjusted for in the Cox model (Table 3, model 2), with a hazard ratio of 0.80 (95% C.I. 0.64–0.98). For sensitivity analysis, we also applied the Cox proportional hazard models to a population matched on individual characteristics, and the results were similar (Supplementary Table S4).

## Discussion

In this retrospective and matched-cohort study, we found PTx to be associated with lower risk of all-cause mortality compared with matched controls not undergoing PTx. Our results corroborate the findings of the large observational study conducted by Kestenbaum *et al.*, in which long-term relative risk of death among PTx patients was 10% to 15% lower than that of matched controls^10^. However, the strength of our conclusions might be greater than that of previous studies for two reasons. First, by restricting enrollment to patients who had undergone a parathyroid scan, we ensured that severe SHPT was present not only in the PTx group but also in the control group. By contrast, the uncertainty of SHPT status in previous large cohort studies might weaken the comparability between the controls and the patients undergoing PTx^10^,^20^. Second, the inclusion of comorbidities in the propensity score and the matching process of our study largely diminished the concern that patients undergoing PTx might be healthier than those not undergoing surgery. This potential selection bias has been acknowledged as the major limitation in previous studies^10^,^20^, and indeed our unmatched PTx patients were less likely to have underlying diabetes mellitus or cardiovascular disease compared with the unmatched controls (Table 1). The matching processes minimized the gap or even reversed the trends of comorbidities between the two groups, and yet the protective effects of PTx remained, consistently suggesting the therapeutic efficacy of PTx for severe SHPT.

Very recently, a nationwide cohort study conducted by Komaba *et al.* also demonstrated a survival benefit of PTx in Japanese hemodialysis patients with severe SHPT^21^. The strengths of this study include its large sample size, matching 4428 PTx patients with the same number of controls, and access to biochemical parameters including iPTH, serum calcium, phosphorus, and albumin. However, the PTx patients in the study were enrolled at the time PTx had been done, thus the clinical data before PTx were unknown. This type of study design may be a source of bias favoring PTx because confounding by indication could not be adequately adjusted for, and mortalities immediately after PTx were not accounted for^22^. Our study complements the study of Komaba *et al.* since our PTx patients were enrolled before surgery. Given that radionuclide parathyroid imaging is usually performed as a pre-PTx routine, both the PTx and non-PTx patients might be referred for the examination under the same indication that their physicians considered them eligible for surgery. With this potential indication and further matching by the propensity score for PTx, our study even more closely approximates the design of a clinical trial, which largely diminishes the confounding by indications. Thus, together with the study of Komaba *et al.*, our results provide further evidence supporting the survival advantage of PTx.

Corroborating the effectiveness of PTx in the treatment of SHPT is of special importance in the era of calcimimetics. Both PTx and calcimimetics aim to improve survival by suppressing iPTH levels, but their efficacies have been questioned due to the potential bias of previous cohort studies and the inconclusive results of the Evaluation of Cinacalcet Hydrochloride Therapy to Lower Cardiovascular Events (EVOLVE) trial^9^,^10^. The intention-to-treat analysis of the EVOLVE trial failed to show a reduction in mortality associated with cinacalcet. However, some authors argued that this trial actually suggested a survival benefit of calcimimetics because its statistical power was only 54% and the *per protocol* lag-censoring analysis showed a significant 17% reduction in mortality^9^,^23^. Our study was minimally confounded by calcimimetics since the approval date of cinacalcet in Taiwan (January 29, 2010) was less than 1 year prior to the end of follow-up. Therefore, in the nationwide cohort, the 20% to 25% reduction in the relative risk of mortality associated with PTx reaffirmed the therapeutic value of lowering iPTH level for SHPT. Whether the same magnitude of efficacy is applicable to calcimimetic agents deserves further investigation.

The exact mechanism by which SHPT increases mortality remains unclear, but predisposition to cardiovascular disorders has been proposed as a possible link^24^. Whether PTx improves survival via reducing cardiovascular risks may not be clearly answered by an observational study like the present one because many cardiovascular risk factors (e.g. age, sex, diabetes mellitus) are themselves associated with increased incidence of PTx^7^,^13^,^14^. Nevertheless, the protective effects of PTx remained significant after adjusting for the adverse impacts of old age, male sex, diabetes mellitus, and cardiovascular diseases including acute myocardial infarction, congestive heart failure, peripheral vascular disease, and cerebrovascular accident (Table 3), implying that PTx may exert its benefits beyond the adjusted factors. To make matters even more complex, we found that two conventional cardiovascular risk factors, hypertension and hyperlipidemia, were paradoxically associated with reduced adverse events (Table 3). This phenomenon was also seen in another NHIRD study^25^ and regarded as a characteristic of the reimbursement data. Since a diagnosis in NHIRD is coded for the claims of specific examinations and/or treatments, patients with the diagnostic code of hypertension and/or hyperlipidemia are usually also prescribed antihypertensive and/or antilipidemic agents. Thus, the significantly lower hazard ratios of these conditions might actually reveal the benefits of associated treatments.

Some limitations in our study should be discussed. First, even though patients with severe SHPT were successfully identified by their history of receiving radionuclide parathyroid imaging, lack of iPTH, serum calcium, and phosphorus data prevented us from performing a detailed comparison of the severity of SHPT or the efficacy of PTx among patients. In addition, the specific use of parathyroid scanning for screening would miss patients with severe SHPT who refused to undergo the imaging survey or received only parathyroid ultrasonography. However, the low sensitivity of screening might be compensated by the high specificity in identifying patients with high iPTH levels and high risk of mortality (Supplementary appendix, Supplement Table S3), the two characteristics of severe SHPT. Furthermore, the sensitivity of parathyroid scanning may still be superior to other potential screening tools in NHIRD, given that parathyroid ultrasonography did not have a specific code until July 1, 2004, and the ICD-9 code for parathyroid disease (252.X) was only present in about half of the scanned patients (data not shown). The competence of radionuclide parathyroid scan for patient selection is further suggested by the fact that PTx patients identified in this way accounts for 1.8% of the eligible ESRD population, a proportion quite similar to the proportion of PTx patients in other ESRD cohorts^10^,^26^,^27^. Second, several important risk factors of mortality in dialysis patients are not available in the NHIRD, such as body mass index, creatinine, serum albumin, and urea clearance. The extensive control of cardiovascular comorbidities in our model might attenuate the confounding effects of these factors, since they usually lead to cardiovascular diseases. However, adjusting for comorbidities can never fully compensate for this limitation. For example, a nearly equal distribution of comorbidities between our matched populations did not guarantee an equal serum albumin level or nutrition status, leaving the possibility that the non-PTx controls might have lower tolerance for the risk of surgery. Unmeasured confounders are the major source of bias, thus our results should be interpreted with caution. Third, conventional medical treatments for SHPT, such as vitamin D sterols and phosphate binders, were not adjusted for in the model owing to the low claim rate in the NHIRD. In other words, these medicines are included in the bundle payment of dialysis, and hence may not be formally recorded. Nevertheless, medical treatments for SHPT were supposed to have failed in our target population; hence the impact of these medicines on clinical outcomes might be relatively small. Finally, the short-term risks of death related to PTx were not evaluated. According to previous studies, the 30-day mortality rate after PTx was approximately 2.0% to 3.1%, but the short-term risk was outweighed by the long-term survival benefits^10^,^28^. The crude mortality rate at the first year after PTx in Taiwan was reported to be 2.9%^13^, which is unlikely to reverse the 20% to 25% mortality-reducing effects observed in the present study.

In conclusion, among a nationwide cohort of dialysis patients who underwent radionuclide parathyroid imaging, we observed a lower long-term risk of death in patients undergoing PTx in comparison with matched controls. These data should be integrated into the clinical decision-making process when treating ESRD patients with unremitting SHPT.

## Methods

### Data Source

National Health Insurance (NHI) is a compulsory, universal health insurance program begun March 1, 1995, that had enrolled over 99% of the 23 million citizens in Taiwan by the end of 2010. The NHI research database (NHIRD) is abstracted from the reimbursement data of the NHI provided by the National Health Insurance Administration, Ministry of Health and Welfare and managed by the National Health Research Institutes for research purposes. The coverage of NHI for the expenditures of patients on maintenance dialysis is comprehensive. Data available in the NHIRD include patient identification numbers, sex, birthdays, dates and specific codes of renal replacement therapy, radionuclide parathyroid imaging (26055A, 26055B), surgical codes of PTx (82007A, 82007B, 82007AA, 82013B), and the ICD-9-CM (International Classification of Disease, 9th Revision, Clinical Modification) diagnostic codes of comorbidities.

### Patient Selection

Patients in the NHIRD who were over 18 years of age and had received maintenance dialysis for more than 3 months in the study period from January 1, 1998 to December 31, 2010 were identified. The criterion of dialysis for more than 3 months was the definition of end-stage renal disease (ESRD) in this study. Since the NHIRD has no laboratory data, to ensure all ESRD patients had high iPTH levels, only those who had received radionuclide parathyroid imaging during the course of maintenance dialysis were enrolled to the cohort. This inclusion criterion guaranteed a high probability of enrolling patients with unremitting SHPT, because in clinical practice, parathyroid scans are usually arranged when a physician fails to control a patient’s SHPT medically and is about to recommend PTx. Exclusion criteria were renal transplantation prior to dialysis or a history of any kind of malignancy before the initiation of long-term dialysis. In addition, patients who remained on a dialysis modality different from the original one for more than 3 months during the study period were considered to demonstrate a switch of dialysis modality and also excluded. In the cohort, patients who had undergone radionuclide parathyroid scanning prior to PTx were classified as the PTx group, while those who had undergone scanning but not PTx served as the control group. In both groups, follow-up began at the time patients underwent radionuclide parathyroid scanning. Patients with a diagnosis of thyroid cancer (ICD-9 code 193), parathyroid cancer (ICD-9 code 194.1), or any kind of head and neck tumor (ICD-9 code 140–149, 160, 161, 235, 210) during the study period were excluded to ensure the parathyroid scans were performed specifically for SHPT. Those who had NHI procedure codes for PTx re-exploration but no preceding codes for PTx or PTx with auto-transplantation were also excluded. For the validation of these patient selection criteria, please see the supplementary information in the online version of this paper. The validation used a single hospital cohort, and the study protocol was approved by the Institutional Review Board (IRB) of E-Da hospital (IRB number: EMRP-103-090). The study was carried out in accordance with the approved guidelines, which authorized us a waiver of the requirements for obtaining informed consent.

### Definition of Mortality

The enrolled patients were considered deceased if any one of the following criteria was met: (1) positive mortality marked in the registration file for catastrophic illness; (2) coding for in-hospital death in the inpatient claim database; and (3) no claim for dialysis or any medical care in the inpatient or outpatient claim database for more than 60 days. The third criterion is a remedy for the imperfect mortality-coding rate of the NHIRD. Given the full coverage of the Taiwanese NHI for all expenditures of dialysis, the possibility of receiving dialysis at one’s own expense in Taiwan is extremely low and negligible. Thus, no claim for any medical care implied a death-related cessation of renal replacement therapy in ESRD patients. Mortality date was determined as either the date of death coding or the date of last dialysis.

### Comorbidity and Propensity Score

The conditions of comorbidities including diabetes (ICD-9 codes 250, 357.2, 362.0X, 366.41, A code 181), hypertension (ICD-9 codes 401–402, 405, A codes 260, 269), hyperlipidemia (ICD-9 codes 272.0–272.4, A code 189), acute myocardial infarction (ICD-9 code 410.X), coronary artery disease (ICD-9 codes 414.8, 414.9), congestive heart failure (ICD-9 codes 398.91, 425, 428, 402.X1, 404.X1, 404.X3), arrhythmia (ICD-9 codes 426–427, V45.0, V53.3), peripheral vascular disease (ICD-9 codes 440–444, 447, 557), cerebral vascular accident (ICD-9 codes 430–438), anemia (ICD-9 codes 280–285), chronic obstructive lung disease (ICD-9 codes 491–494, 496), gastrointestinal bleeding (ICD-9 codes 456.0–456.2, 530.7, 531–534, 569.84, 569.85, 578), liver disease (ICD-9 codes 070, 570, 571, 572.2–572.4, 573.1–573.3), and dementia (ICD-9 code: 290) were obtained from both inpatient and outpatient reimbursement data. To avoid inclusion of miscoded outpatients, a comorbidity was confirmed in the outpatient claim database only when its codes were present three or more times within 12 months, with the first and last outpatient visit at least 30 days apart. On the other hand, comorbidity identification in the hospitalization database requires the presence of the codes only once^25^,^29^. The comorbidities were recorded in two divided periods. The first was the 1-year period prior to parathyroid scan. Comorbidities in this period in addition to age, sex, dialysis modality, and dialysis duration at the time of scan were risk factors for PTx^7^,^11^,^12^,^13^,^14^, and hence were taken into account in the logistic regression model for calculating propensity score, estimating the conditional probability of being assigned to the PTx group. The second period was defined as the time from parathyroid scan to the time of being censored or to the end of the study. Comorbidities in this period as well as the PTx procedure were modeled as time-dependent covariates in the multivariate Cox proportional hazard model for all-cause mortality.

### Statistical Analyses

Results were expressed as mean ± standard deviation for continuous variables and as percentage for categorical variables. The mortality rate was calculated by dividing the number of deaths by the number of person-years at risk. To minimize potential confounding, the PTx and control groups were matched based on the propensity score for PTx to achieve a 1:1 case-control match. The matching process used a greedy algorithm^30^. Standardized difference, calculated as the difference in proportions or means divided by a pooled estimate of standard deviation, was utilized to assess the balance of covariates between propensity score-matched groups. The Cox proportional hazard models were applied to the matched populations to evaluate the association of PTx with all-cause mortality. Two kinds of Cox model were constructed for each matched population. Model 1 was adjusted for the matching covariates before parathyroid scan to account for potential residual differences in confounding from the matching processes. Model 2 was adjusted for comorbidities before and after scanning, whereas the comorbidities after scanning were set as time-dependent covariates. In both models, PTx was set as a time-dependent covariate to avoid the immortal time bias^31^, and age, sex, dialysis modality, and dialysis duration at the time of scan were all adjusted. Survival time began from the first date of parathyroid scan and was censored if the patient received renal transplantation, died, or reached the end of the study period. The data were analyzed using the SAS system for Windows, version 9.3, statistical analysis software (SAS Institute, Inc., Cary, NC). For all statistical testing, a two-sided P value < 0.05 indicated a statistically significant result.

## Additional Information

**How to cite this article**: Ho, L.-C. *et al.* Parathyroidectomy Associates with Reduced Mortality in Taiwanese Dialysis Patients with Hyperparathyroidism: Evidence for the Controversy of Current Guidelines. *Sci. Rep.* **6**, 19150; doi: 10.1038/srep19150 (2016).

## Supplementary Material

Supplementary Information

## Figures and Tables

**Table 1:** Baseline characteristics of the dialysis patients with or without parathyroidectomy (PTx) at the time of undergoing radionuclide parathyroid scanning, before and after being matched on propensity score.

	Before matching			After matching		
	Overall (n = 2786)	PTx (n = 1707)	Control (n = 1079)	PTx (n = 998)	Control (n = 998)	Standardized difference§
Age (years)	54.0 ± 12.5	52.9 ± 11.9	55.8 ± 13.4	54.7 ± 11.8	55.0 ± 13.1	0.02
Male sex (%)	1131(40.6)	676 (39.6)	455 (42.2)	428 (42.9)	424 (42.5)	0.01
HD (%)	2548 (91.5)	1577 (92.4)	971 (90.0)	902 (90.4)	903 (90.5)	<0.01
Dialysis duration (years)	4.61 ± 2.98	4.90 ± 2.93	4.14 ± 3.00	4.24 ± 2.78	4.31 ± 2.99	0.02
DM (%)	604 (21.7)	346 (20.3)	258 (23.9)	244 (24.5)	219 (21.9)	0.06
Hypertension (%)	1434 (51.5)	859 (50.3)	575 (53.3)	539 (54.0)	518 (51.9)	0.04
Hyperlipidemia (%)	680 (24.4)	439 (25.7)	241 (22.3)	279 (28.0)	213 (21.3)	0.15
AMI (%)	71 (2.6)	32 (1.9)	39 (3.6)	24 (2.4)	29 (2.9)	0.03
CAD (%)	396 (14.2)	234 (13.7)	162 (15.0)	173 (17.3)	137 (13.7)	0.10
CHF (%)	448 (16.1)	244 (14.3)	204 (18.9)	179 (17.9)	181 (18.1)	0.01
Arrhythmia (%)	327 (11.7)	184 (10.8)	143 (13.3)	134 (13.4)	124 (12.4)	0.03
PVD (%)	399 (14.3)	258 (15.1)	141 (13.1)	167 (16.7)	125 (12.5)	0.12
CVA (%)	308 (11.1)	177 (10.4)	131 (12.1)	134 (13.4)	118 (11.8)	0.05
Anemia¶ (%)	463 (16.6)	233 (13.7)	230 (21.3)	179 (17.9)	184 (18.4)	0.01
COPD (%)	282 (10.1)	154 (9.0)	128 (11.9)	97 (9.7)	116 (11.6)	0.06
GI bleeding (%)	725 (26.0)	476 (27.9)	249 (23.1)	302 (30.3)	232 (23.3)	0.16
Liver disease (%)	558 (20.0)	339 (19.9)	219 (20.3)	212 (21.2)	189 (18.9)	0.06
Dementia (%)	33 (1.2)	14 (0.8)	19 (1.8)	13 (1.3)	14 (1.4)	0.01

Abbreviations: HD, hemodialysis; DM, diabetes mellitus; AMI, acute myocardial infarction; CAD, coronary artery disease; CHF, congestive heart failure; PVD, peripheral vascular disease; CVA, cerebral vascular accident, COPD, chronic obstructive pulmonary disease; GI bleeding, gastrointestinal bleeding; PTx, parathyroidectomy.

^§^Standardized difference = difference in proportions or means divided by a pooled estimate of standard deviation.

^¶^May only indicate resistance of erythropoiesis-stimulating agents and/or requirement of blood transfusion (supplementary information).

**Table 2:** Accumulated person-years, mean follow-up time, and crude mortality rate in dialysis patients with or without parathyroidectomy (PTx).

	Before matching			After matching	
	Overall (n = 2786)	PTx (n = 1707)	Control (n = 1079)	PTx (n = 998)	Control (n = 998)
Person-years	8570	5746	2824	3450	2641
Mean follow-up time (years)	3.08 ± 2.44	3.37 ± 2.47	2.62 ± 2.32	3.46 ± 2.51	2.65 ± 2.35
Overall death (%)	492 (17.7)	234 (13.7)	258 (23.9)	162 (16.2)	224 (22.4)
Crude mortality rate (per 10,000 person-years)	574	407	914	470	848

**Table 3:** Hazard ratios (HR) and 95% confidence intervals (C.I.) for Cox proportional hazard models predicting all-cause mortality, adjusted for comorbidities present before radionuclide parathyroid scan or in the whole study period.

	Model 1#		Model 2§	
	HR	95% C.I.	HR	95% C.I.
Parathyroidectomy	0.76^**^	0.61–0.94	0.80^*^	0.64–0.98
Age (for every 1-year increase)	1.05^***^	1.04–1.06	1.04^***^	1.03–1.05
Sex (male vs. female)	1.24^*^	1.00–1.53	1.28^*^	1.04–1.58
Dialysis modality (HD vs. PD)	0.77	0.48–1.26	0.77	0.47–1.24
Dialysis duration (for every 1-year increase)	1.03	0.99–1.08	1.03	0.99–1.07
DM	1.58^***^	1.27–1.97	1.80^***^	1.43–2.26
Hypertension	0.62^***^	0.50–0.76	0.66^***^	0.54–0.82
Hyperlipidemia	0.62^***^	0.48–0.80	0.71^**^	0.55–0.92
AMI	1.31	0.83–2.07	2.21^***^	1.38–3.53
CAD	1.04	0.80–1.36	1.05	0.81–1.37
CHF	1.09	0.85–1.40	1.29^*^	1.01–1.66
Arrhythmia	0.93	0.71–1.21	1.20	0.92–1.57
Peripheral vascular disease	1.17	0.91–1.49	1.60^***^	1.25–2.04
CVA	1.50^**^	1.16–1.92	1.80^***^	1.40–2.33
Anemia¶	0.82	0.62–1.09	0.97	0.73–1.28
COPD	0.83	0.62–1.11	0.90	0.67–1.21
GI bleeding	0.89	0.71–1.11	1.18	0.94–1.48
Liver disease	0.96	0.75–1.23	1.15	0.90–1.46
Dementia	1.24	0.66–2.34	1.38	0.73–2.62

*P ≤ 0.05, **P ≤ 0.01, ***P ≤ 0.001

Abbreviations: HD, hemodialysis; PD, peritoneal dialysis; DM, diabetes mellitus; AMI, acute myocardial infarction; CAD, coronary artery disease; CHF, congestive heart failure; PVD, peripheral vascular disease; CVA, cerebral vascular accident, COPD, chronic obstructive pulmonary disease; GI bleeding, gastrointestinal bleeding.

^#^Model 1 was adjusted for comorbidities before radionuclide parathyroid imaging.

^§^Model 2 was adjusted for comorbidities before and after radionuclide parathyroid imaging.

^¶^May only indicate resistance of erythropoiesis-stimulating agents and/or requirement of blood transfusion (supplementary information).
