# Supplementary material for: Parathyroidectomy Associates with Reduced Mortality in Taiwanese Dialysis Patients with Hyperparathyroidism: Evidence for the Controversy of Current Guidelines
Source: Sci Rep. 2016 Jan 13;6:19150. doi: 10.1038/srep19150 (PMC4725823; doi:10.1038/srep19150)
Supplement: Supplementary Information [file srep19150-s1.doc]

**Supplementary Information**

**Parathyroidectomy Associates with Reduced Mortality in Taiwanese Dialysis Patients with Hyperparathyroidism: Evidence for the Controversy of Current Guidelines**

Li-Chun Ho^1,2^, Shih-Yuan Hung^2^, Hsi-Hao Wang^2^, Te-Hui Kuo^3,4^, Yu-Tzu Chang^1,3^, Chin-Chung Tseng^3^, Jia-Ling Wu^3^, Chung-Yi Li^5,6^, Jung-Der Wang^3,5,7^, Yau-Sheng Tsai^1^, and Junne-Ming Sung^3,*^ for the Tainan RENal Disease Study (TRENDS) group

^1^Graduate Institute of Clinical Medicine, National Cheng Kung University, Tainan; ^2^Division of Nephrology, Department of Internal Medicine, E-Da Hospital/I-Shou University, Kaohsiung; ^3^Department of Internal Medicine, National Cheng Kung University Medical College and Hospital, Tainan; ^4^Graduate Institute of Public Health, ^5^Department of Public Health, National Cheng Kung University College of Medicine, Tainan; ^6^Department of Public Health, College of Public Health, China Medical University, Taichung; ^7^Department of Occupational and Environmental Medicine, National Cheng Kung University Hospital, Tainan, Taiwan.

*Patients without severe SHPT*

The study cohort derived from NHIRD in the period from January 1, 1998 to December 31, 2010 is described in the “**Methods**” including maintenance dialysis patients over 18 years of age and excluding those with dialysis modality switched during the study period, renal transplantation, or a diagnosis of malignancy prior to long-term dialysis. In this cohort, a patient was considered not to have severe SHPT when he or she had no ICD-9 codes corresponding to parathyroid disease (252.X) and had not undergone any one of the following procedures: radionuclide parathyroid scan, parathyroid ultrasonography, or PTx. However, since there was no specific NHIRD code for parathyroid ultrasonography until July 1, 2004, we could not exclude patients with parathyroid ultrasonography prior to that date. Patients who fulfilled the above criteria were classified as the non-scan group and were compared with those who had undergone a parathyroid scan but not PTx, namely the scan group. The conditions of comorbidities were the same as those described in the “**Methods**” but the time reference was the time of dialysis initiation instead of the time of the first parathyroid scan. Comorbidities in the 1-year period before maintenance dialysis in addition to age, sex, and dialysis modality were used to calculate the propensity score, estimating the conditional probability of being assigned to parathyroid scan. The scan and non-scan patients were then 1:3 matched based on propensity score. The multivariate Cox proportional hazard model was applied to the matched populations to evaluate the association of parathyroid scanning with all-cause mortality. The parathyroid scan was set as a time-dependent covariate in the Cox proportional hazard model. Comorbidities before and after the initiation of dialysis were also adjusted in the model, whereas comorbidities after initiating dialysis were modeled as time-dependent covariates.

*Patients with radionuclide parathyroid imaging in a single-hospital cohort*

The patient selection criteria described in “**Methods**” was applied to the cohort of E-Da hospital. E-Da hospital began operation in April 2004 and has been accredited as a regional teaching hospital since August 2004. A total of 115 maintenance dialysis patients over 18 years of age underwent radionuclide parathyroid scanning at E-Da hospital during the period from August 1, 2004 to December 31, 2010. Among them, 6 (5.2%) patients were excluded because of a diagnosis of malignancy before the initiation of dialysis, 3 (2.6%) because of renal transplantation prior to regular dialysis, and 6 (5.2%) because of dialysis modality switch for more than 3 months during the study period. Of the remaining 100 patients, further exclusion was made for 1 (1%) undergoing PTx before the initiation of regular dialysis and 4 (4%) undergoing PTx before parathyroid scan. Finally, 95 scanned patients were eligible for analysis. Of these patients, 10 were on peritoneal dialysis, 64 had undergone PTx, and 37 were referred from outlying hospitals or clinics. The serum levels of intact parathyroid hormone (iPTH) nearest to the date of parathyroid scan but prior to PTx were recorded. The study protocol was approved by the Institutional Review Board (IRB) of E-Da hospital (IRB number: EMRP-103-090). The study was carried out in accordance with the approved guidelines, which authorized us a waiver of the requirements for obtaining informed consent.

*Validation of radionuclide parathyroid imaging as an indicator of severe SHPT*

To validate the accuracy of parathyroid scanning as a selection criterion for patients with severe SHPT, we first examined iPTH levels of the 95 scanned dialysis patients from a single hospital. Serum iPTH levels were available in 92 patients. All the measurements were made within 3 months of parathyroid scanning except one that took place 4 months before the scan. The median iPTH concentration was 1146.6 pg/mL, and the lowest recorded concentration was 415.7 pg/mL. Seventy-four (80.4%) patients had an iPTH concentration exceeding 800 pg/mL, and the 9 (9.8%) patients with iPTH levels less than 600 pg/mL all had hypercalcemia and/or a serum phosphorus level above 5.5 mg/dL.

Comparison between the scan and non-scan groups from the NHIRD provided further validation. Before matching, the scanned patients tended to be younger, with a lower proportion male, and were much less likely to have diabetes mellitus than the patients without parathyroid scan (Table S1). After being matched by propensity score for parathyroid scan, the scan and the non-scan group demonstrated equal distributions in age, sex, dialysis modality, and diabetes mellitus, but the proportions of other comorbidities were higher in the scan group than in the non-scan group (Table S1). The mortality rate was lower in the scan group than in the non-scan group even after the matching process (Table S2), yet the Cox proportional hazard model for all-cause mortality revealed that parathyroid scanning was associated with increased mortality, with a hazard ratio of 1.46 (95% C.I. 1.26–1.69) (Table S3). These results suggest that parathyroid scanning is related to high iPTH levels and high risk of death, and hence is an adequate indicator for severe SHPT.

*Sensitivity analysis in a population matched on individual characteristics*

For sensitivity analysis, another kind of matching was applied to the patients who had received radionuclide parathyroid imaging. In this kind of matching, each control was individually matched to each PTx patient by age (±2 years), sex, dialysis modality, dialysis duration (± 1 year), and diabetes status at the time of parathyroid scan. A total of 905 PTx patients were matched to the same number of controls, and the crude mortality rates were 441/10,000 person-years and 797/10,000 person-years respectively. The Cox proportional hazard model for all-cause mortality, adjusted for comorbidities before scanning (model 1) or in the whole study period (model 2), was applied to this population matched on individual characteristics. The results were shown in Table S4. PTx was associated with a reduction of all-cause mortality in both models (model 1: hazard ratio = 0.74, 95% C.I. 0.59–0.92; model 2: hazard ratio = 0.78, 95% C.I. 0.62–0.97). The result of the sensitivity analysis suggests the robustness of our conclusion that PTx is an effective therapy for dialysis patients with severe SHPT.

*The proportion of patients underwent parathyroid auto-transplantation*

In the NHIRD, ‘PTx’ and ‘PTx with auto-transplantation’ are coded in two different kinds of procedure codes, but there are no specific codes for ‘subtotal PTx’ and ‘total PTx’. Among the patients who received parathyroid scan and underwent PTx (n = 1707), 183 (10.7%) underwent auto-transplantation while the other 1524 (89.3%) did not. Since total PTx is usually accompanied with auto-transplantation while subtotal PTx is not, we assume that the codes for ‘PTx with auto-transplantation’ actually mean ‘total PTx with auto-transplantation’ while the codes for ‘PTx’ actually mean ‘subtotal PTx’. If so, the preferred procedure of PTx in Taiwan in the study period from 1998 to 2010 was subtotal PTx.

*Probable characteristics of dialysis patients coded with anemia in the NHIRD*

According to the bundled payment system for dialysis patients in Taiwan, ICD-9 codes of anemia are not necessary for the reimbursement of erythropoiesis-stimulating agents (ESAs) but necessary for the reimbursement of blood transfusion. Therefore, the patients with the codes of anemia in our study were probably those with severe, ESA-resistant anemia requiring blood transfusion instead of those with stable hemoglobin levels requiring merely regular doses of ESAs. This might explain why only 16.6% of the enrolled dialysis patients were coded with the ICD-9 codes of anemia (Table 1).

**Table S1. Baseline characteristics of the dialysis patients with or without radionuclide parathyroid scan at the time of initiating maintenance dialysis, before and after propensity score matching**

|  | **Before matching** | | **Matched on propensity score** | |
| --- | --- | --- | --- | --- |
|  | **Scan**  **(n = 1079)** | **Non-scan**  **(n = 81991)** | **Scan**  **(n = 1079)** | **Non-scan**  **(n = 3237)** |
| **Age (years)** | 51.6 ± 13.5 | 62.7 ± 13.9 | 51.6 ± 13.5 | 51.6 ± 13.8 |
| **Male sex (%)** | 455 (42.2) | 41704 (50.9) | 455 (42.2) | 1386 (42.8) |
| **HD (%)** | 971 (90.0) | 77069 (94.0) | 971 (90.0) | 2936 (90.7) |
| **DM (%)** | 298 (27.6) | 45776 (55.8) | 298 (27.62) | 879 (27.15) |
| **Hypertension (%)** | 841 (77.9) | 62912 (76.7) | 841 (77.9) | 2329 (72.0) |
| **Hyperlipidemia (%)** | 391 (36.2) | 21224 (25.9) | 391 (36.2) | 770 (23.8) |
| **AMI (%)** | 55 (5.1) | 4617 (5.6) | 55 (5.1) | 82 (2.5) |
| **CAD (%)** | 243 (22.5) | 15089 (18.4) | 243 (22.5) | 427 (13.2) |
| **CHF (%)** | 331 (30.7) | 23629 (28.8) | 331 (30.7) | 637 (19.7) |
| **Arrhythmia (%)** | 191 (17.7) | 9748 (11.9) | 191 (17.7) | 305 (9.4) |
| **PVD (%)** | 212 (19.7) | 12600 (15.4) | 212 (19.7) | 422 (13.0) |
| **CVA (%)** | 189 (17.5) | 20827 (25.4) | 189 (17.5) | 504 (15.6) |
| **Anemia (%)** | 451 (41.8) | 23099 (28.2) | 451 (41.8) | 1068 (33.0) |
| **COPD (%)** | 204 (18.9) | 12661 (15.4) | 204 (18.9) | 362 (11.2) |
| **GI bleeding (%)** | 403 (37.4) | 26402 (32.2) | 403 (37.4) | 952 (29.4) |
| **Liver disease (%)** | 296 (27.4) | 13970 (17.0) | 296 (27.4) | 664 (20.5) |
| **Dementia (%)** | 28 (2.6) | 3554 (4.3) | 28 (2.6) | 55 (1.7) |

Abbreviations: HD, hemodialysis; DM, diabetes mellitus; AMI, acute myocardial infarction; CAD, coronary artery disease; CHF, congestive heart failure; PVD, peripheral vascular disease; CVA, cerebral vascular accident, COPD, chronic obstructive pulmonary disease; GI bleeding, gastrointestinal bleeding.

**Table S2. Accumulated person-years, mean follow-up time, and crude mortality rates in dialysis patients with or without radionuclide parathyroid scan, before and after propensity score matching**

|  | **Before matching** | | **Matched on propensity score** | |
| --- | --- | --- | --- | --- |
|  | **Scan**  **(n = 1079)** | **Non-scan**  **(n = 81991)** | **Scan**  **(n = 1079)** | **Non-scan**  **(n = 3237)** |
| **Person-years** | 7274 | 280342 | 7274 | 15033 |
| **Mean follow-up time (years)** | 6.74 ± 3.12 | 3.42 ± 2.95 | 6.74 ± 3.12 | 4.64 ± 3.40 |
| **Overall death (%)** | 258 (23.9) | 41584 (50.7) | 258 (23.9) | 1050 (32.4) |
| **Crude mortality rate (per 10,000 person-years)** | 355 | 1484 | 355 | 698 |

**Table S3. Hazard ratios (HR) and 95% confidence intervals (C.I.) for Cox proportional hazard models predicting all-cause mortality risk, adjusted for comorbidities before and after initiating maintenance dialysis, in a dialysis population with or without radionuclide parathyroid scan**

|  | HR | 95% C.I. | *P* |
| --- | --- | --- | --- |
| **Scan** | **1.46** | 1.26–1.69 | < 0.001 |
| Age (for every 1 year increase) | 1.04 | 1.04–1.05 | < 0.001 |
| Sex (male vs. female) | 1.18 | 1.06–1.32 | 0.003 |
| Dialysis modality (HD vs. PD) | 0.78 | 0.59–1.03 | 0.077 |
| DM | 1.70 | 1.51–1.92 | < 0.001 |
| Hypertension | 0.73 | 0.65–0.83 | < 0.001 |
| Hyperlipidemia | 0.77 | 0.67–0.88 | < 0.001 |
| AMI | 2.00 | 1.55–2.57 | < 0.001 |
| CAD | 0.88 | 0.76–1.03 | 0.104 |
| CHF | 1.39 | 1.22–1.59 | < 0.001 |
| Arrhythmia | 1.07 | 0.91–1.26 | 0.420 |
| Peripheral vascular disease | 1.06 | 0.91–1.23 | 0.478 |
| CVA | 1.95 | 1.71–2.23 | < 0.001 |
| Anemia | 0.85 | 0.76–0.96 | 0.008 |
| COPD | 1.02 | 0.88–1.18 | 0.777 |
| GI bleeding | 1.22 | 1.08–1.38 | 0.001 |
| Liver disease | 1.24 | 1.09–1.41 | 0.001 |
| Dementia | 1.12 | 0.82–1.53 | 0.488 |

Abbreviations: HD, hemodialysis; PD, peritoneal dialysis; DM, diabetes mellitus; AMI, acute myocardial infarction; CAD, coronary artery disease; CHF, congestive heart failure; CVA, cerebral vascular accident, COPD, chronic obstructive pulmonary disease; GI bleeding, gastrointestinal bleeding.

**Table S4. Hazard ratios (HR) and 95% confidence intervals (C.I.) for Cox proportional hazard models predicting all-cause mortality, adjusted for comorbidities present before radionuclide parathyroid scan or in the whole study period, in a parathyroid scanned population matched on individual characteristics**

|  | **Model 1**^#^ | | **Model 2**^§^ | |
| --- | --- | --- | --- | --- |
|  | **HR** | **95% C.I.** | **HR** | **95% C.I.** |
| **Parathyroidectomy** | **0.74^**^** | **0.59–0.92** | **0.78^*^** | **0.62–0.97** |
| Age (for every 1-year increase) | 1.05^***^ | 1.04–1.06 | 1.04^***^ | 1.03–1.05 |
| Sex (male vs. female) | 1.21 | 0.97–1.52 | 1.24 | 0.99–1.56 |
| Dialysis modality (HD vs. PD) | 0.70 | 0.37–1.32 | 0.68 | 0.36–1.27 |
| Dialysis duration (for every 1-year increase) | 1.03 | 0.98–1.07 | 1.02 | 0.98–1.07 |
| DM | 1.76^***^ | 1.38–2.23 | 2.06^***^ | 1.61–2.63 |
| Hypertension | 0.65^***^ | 0.51–0.81 | 0.67^***^ | 0.53–0.84 |
| Hyperlipidemia | 0.61^***^ | 0.46–0.80 | 0.69^**^ | 0.52–0.91 |
| AMI | 1.15 | 0.70–1.88 | 1.89^*^ | 1.14–3.13 |
| CAD | 0.99 | 0.74–1.33 | 1.01 | 0.76–1.35 |
| CHF | 1.04 | 0.79–1.37 | 1.25 | 0.95–1.64 |
| Arrhythmia | 1.04 | 0.78–1.39 | 1.33 | 0.99–1.77 |
| Peripheral vascular disease | 1.13 | 0.87–1.48 | 1.56^***^ | 1.19–2.04 |
| CVA | 1.44^**^ | 1.09–1.91 | 1.75^***^ | 1.32–2.31 |
| Anemia | 0.81 | 0.60–1.09 | 0.97 | 0.72–1.30 |
| COPD | 0.75 | 0.54–1.03 | 0.83 | 0.60–1.15 |
| GI bleeding | 0.95 | 0.75–1.21 | 1.19 | 0.94–1.52 |
| Liver disease | 0.93 | 0.72–1.20 | 1.17 | 0.91–1.51 |
| Dementia | 1.54 | 0.82–2.89 | 2.01^*^ | 1.06–3.80 |

^*^ P ≤ 0.05, ^**^ P ≤ 0.01, ^***^ P ≤ 0.001

^#^ Model 1 was adjusted for comorbidities before radionuclide parathyroid imaging.

^§^ Model 2 was adjusted for comorbidities before and after radionuclide parathyroid imaging.

Abbreviations: HD, hemodialysis; PD, peritoneal dialysis; DM, diabetes mellitus; AMI, acute myocardial infarction; CAD, coronary artery disease; CHF, congestive heart failure; PVD, peripheral vascular disease; CVA, cerebral vascular accident, COPD, chronic obstructive pulmonary disease; GI bleeding, gastrointestinal bleeding.
